# Finding solace in the ocean

**DOI:** 10.1177/26323524251372968

**Published:** 2025-09-01

**Authors:** Maja Furlan de Brito

**Affiliations:** 1 Faculty of Medicine, University of Coimbra, Portugal; 2 Centre for Innovative Biomedicine and Biotechnology, University of Coimbra, Portugal; 3 Cicely Saunders Institute of Palliative Care, Policy & Rehabilitation, King’s College London, UK

**Keywords:** grief, nature, essay

## Abstract

This is a narrative essay about what the ocean taught me about my personal grief, lessons I, as surprisingly as it may seem, never learned through advanced training in bereavement support.

It was about 2 years after my husband’s death, and I was standing at the waterline on the beach at Gigi, a Portuguese surf paradise, with my longboard in my hand, feeling equally strong forces calling me in and keeping me out at the same time. All that was moving were tears rolling down as if they wanted to connect me with the ocean from afar. I was standing still, paralyzed between the fear of losing control and the comforting melody of the ocean calling my name. “Come, come inside, let the sorrow melt with the gentle rocking and the mellow waves.” “Don’t, don’t go inside! Darkness may prevail if you go inside. Dark and bitter anger may find its way out and drown you.” This would have been my first surf in 10 years.

I needed someone to walk with me on this adventure, freeing me from the cold embrace of my frozen-like state. So, a month later, I began surf classes with a surf coach. It was an internal battle between the ocean’s destructive power of seeing and hearing the screams of the dark monsters coming from my deepest parts, and the desire of catching and riding a small wave. Some to the right, some to the left, and being left feeling like “a happy turtle on a rainbow.” Relaxed and playful, with a new zest for the turning of pages of my upcoming chapters.

The struggle felt constant, though. Shifting toward the dark side happened more often than toward the rainbow bliss. Cami, my coach, would regularly give feedback that I’m good at timing, that once I do catch a wave, my take-off was good, that for someone who just started, I read the waves well enough and was progressing quickly. Yet, I could not catch the wave on my own; I needed her push every time. I once messaged her when leaving my office at the end of the day, asking if she had been messing with me and giving me positive feedback just to make me feel good about myself, and to be more relaxed, or was my surfing really was all that she had been saying. To this day, I remember where I was walking, what I was wearing, how the world smelled that evening when I realized I had everything I needed inside of me. Supposing the conditions were optimal and fitted to my level, the catching of the wave, the take-off, and enjoying the ride, it very much depended on me.

For some reason, it seemed like I didn’t want to catch the wave. What waves wouldn’t I want to catch? Cami would often point out that my paddling lacked conviction. Some surfers passing by would agree and make comments like: “It looks like you are not sure whether you actually want to surf. Do you want to catch the wave or not?”

Do I? Good question. Do I want to return to and continue on in this world where two of my dearest people do not exist anymore? I lost my mum to cancer some years ago, as well as my husband. Do I want to go on in a world of mess left behind after the death of my husband? In a world of work delays that have accumulated through the years of cancer, COVID, and personal despair, to a point where I have forgotten how to start digging in again? In a world that has become too unbearable to be carried on the shoulders of one person. In a world that did not feel safe and was full of uncertainty. In a world where things I had been doing and ways I had been living had made life stop making sense to me? In a world that seemed to be telling me that I should have been “back to normal” again by then, by now.

It became a question of: do I want to make the next step forward? And how to do it. Do I want to face the mess this life has turned into? What happens if you find yourself in the impact zone and paddle away from it quick enough? And what happens if you don’t paddle against or with the wave when you see it traveling toward you? That meant starting to paddle with conviction, taking off, communicating my wishes, marking my space, and, once standing up, opening my chest and decisively pointing in the direction I wanted to ride. Easier said than done.

Spontaneously, over time, I developed a ritual. After the session, I would take the upper part or the whole wetsuit off and return to the water bare skin. Almost as if letting the water flow through me. I’d say to the ocean, “wash off what is ready to go away, cleanse what is here to stay.” I would surrender myself completely to the ocean, letting it move my body as it wished. Sometimes a gentle lap, other times a hard crush against my chest or back. For that moment, the weight of everything that hurt was lifted. It was in those minutes that I felt the totality of my loss be released in all its brutality and in all its beauty at the same time. In the safe space of the ocean, in the silence of the underwater world, where no one could judge it, tell me how to feel or express it. How to go about it. Where the pressures of time and space do not exist. It was just me in a cold, yet comforting embrace of the ocean. The ocean never had the intent to mold my grief; rather, it provided a safe and accepting space for it to be held. Slowly, this twosome of me and the ocean became a threesome. A new vision for the future was joined.

It took me time. By this society’s standards, it took me too long of a time. Being with grief in the ocean, I found acceptance of how much it hurt, how long it has been taking, and how confused life became. Most of all, I found acceptance for all the opportunities I have been feeling like a lost one because of my twists and turns. Despite its ferocious energy, in it, I found comfort and reassurance in my attempts of taking off time and time again. I found ways of carrying on despite churning waves and whipping winds, making me stumble repeatedly.

The ocean taught me things about grief that my years-long psychotherapeutic and advanced grief therapy training did not. The importance of grief simply being held, not even explained, let alone being intervened. The importance of creating a safe space where grief can reside with us. Through contact with nature, through movement, through breathing, through just being. Until it becomes a written word . . .

